# Interventions to increase facility births and provision of postpartum care in sub-Saharan Africa: a scoping review

**DOI:** 10.1186/s12978-021-01072-4

**Published:** 2021-01-21

**Authors:** Bienvenu Salim Camara, Alexandre Delamou, Fassou Mathias Grovogui, Bregje Christina de Kok, Lenka Benova, Alison Marie El Ayadi, Rene Gerrets, Koen Peeters Grietens, Thérèse Delvaux

**Affiliations:** 1 Department of Public Health, Institute of Tropical Medicine, Nationalestraat 155, 2000 Antwerp, Belgium; 2 Department of Anthropology, Amsterdam Institute of Social Science Research, Amsterdam, The Netherlands; 3 Centre National de Formation Et de Recherche en Santé Rurale de Maferinyah, Forécariah, Guinea; 4 Centre D’Excellence Africain Pour La Prévention Et Le Contrôle Des Maladies Transmissibles (CEA-PCMT), Conakry, Guinea; 5 Department of Obstetrics, Gynecology and Reproductive Sciences, Bixby Center for Global Reproductive Health, University of California, San Francisco, CA USA

**Keywords:** Facility birth, Postpartum care, Intervention, Sub-saharan africa, Scoping review

## Abstract

**Background:**

Most maternal deaths occur during the intrapartum and peripartum periods in sub-Saharan Africa, emphasizing the importance of timely access to quality health service for childbirth and postpartum care. Increasing facility births and provision of postpartum care has been the focus of numerous interventions globally, including in sub-Saharan Africa. The objective of this scoping review is to synthetize the characteristics and effectiveness of interventions to increase facility births or provision of postpartum care in sub-Saharan Africa.

**Methods:**

We searched for systematic reviews, scoping reviews, qualitative studies and quantitative studies using experimental, quasi experimental, or observational designs, which reported on interventions for increasing facility birth or provision of postpartum care in sub-Saharan Africa. These studies were published in English or French. The search comprised six scientific literature databases (Pubmed, CAIRN, la Banque de Données en Santé Publique, the Cochrane Library). We also used Google Scholar and snowball or citation tracking.

**Results:**

Strategies identified in the literature as increasing facility births in the sub-Saharan African context include community awareness raising, health expenses reduction (transportation or user fee), non-monetary incentive programs (baby kits), or a combination of these with improvement of care quality (patient’s privacy, waiting time, training of provider), and or follow-up of pregnant women to use health facility for birth. Strategies that were found to increase provision of postpartum care include improvement of care quality, community-level identification and referrals of postpartum problems and transport voucher program.

**Conclusions:**

To accelerate achievements in facility birth and provision of postpartum care in sub-Saharan Africa, we recommend strategies that can be implemented sustainably or produce sustainable change. How to sustainably motivate community actors in health interventions may be particularly important in this respect. Furthermore, we recommend that more intervention studies are implemented in West and Central Africa, and focused more on postpartum.

**Plain English summary:**

In in sub-Saharan Africa, many women die when giving or few days after birth. This happens because they do not have access to good health services in a timely manner during labor and after giving birth. Worldwide, many interventions have been implemented to Increase the number of women giving birth in a health facility or receiving care from health professional after giving birth. The objective of this study is to synthetize the characteristics and effectiveness of interventions that have been implemented in sub-Saharan Africa, aiming to increase the number of women giving birth in a health facility or receiving care from health professional after birth. To proceed with this synthesis, we did a review of studies that have reported on such interventions in sub-Saharan Africa. These studies were published in English or French. The interventions identified to increase the number of women giving birth in a health facility include community awareness raising, reduction of health expenses (transportation or user fee), non-monetary incentive programs (baby kits), or a combination of these with improvement of care quality (patient’s privacy, waiting time, training of provider), and or follow-up of pregnant women to use health facility for birth. Interventions implemented to increase the number women receiving care from a health professional after birth include improvement of care quality, transport voucher program and community-level identification and referrals to the health center of mothers’ health problems. In sub-Saharan Africa, to accelerate increase in the number of women giving birth in a health facility and receiving care from a health professional after, we recommend interventions that can be implemented sustainably or produce sustainable change. How to sustainably motivate community actors in health interventions may be particularly important in this respect. Furthermore, we recommend the conduct in West and Central Africa, of more studies targeting interventions to increase the number of women giving birth in a health facility and or receiving care from a health professional after birth.

## Résumé

**Contexte:**

La plupart des décès maternels surviennent pendant les périodes intrapartum et péripartum en Afrique subsaharienne, ce qui souligne l'importance de l’accès à temps aux services de santé de qualité pour l'accouchement et les soins post-partum. L'augmentation des accouchements institutionnels et l’offre de soins post-partum ont fait l'objet de nombreuses interventions dans le monde entier, y compris en Afrique subsaharienne. L'objectif de cette étude est de synthétiser les caractéristiques et l'efficacité des interventions visant à accroître les accouchements institutionnels ou l’offre de soins post-partum en Afrique subsaharienne.

**Méthodes:**

Nous avons recherché des revues systématiques, des revues de portée, des études qualitatives et des études quantitatives utilisant des types expérimentaux, quasi expérimentaux ou d'observation, qui rapportaient sur des interventions visant à accroître les accouchements institutionnels et l’offre de soins post-partum en Afrique subsaharienne. Ces études ont été publiées en anglais ou en français. La recherche a porté sur six bases de données de littérature scientifique (Pubmed, CAIRN, la Banque de Données en Santé Publique, la Cochrane Library). Nous avons également utilisé Google Scholar et le suivi des boules de neige ou des citations.

**Résultats:**

Les stratégies identifiées dans la littérature comme accroissant les accouchements intentionnels dans le contexte de l'Afrique subsaharienne comprennent la sensibilisation des communautés, la réduction des dépenses de santé (transport ou frais d'utilisation), des programmes d'incitation non monétaires (kits pour bébés), ou une combinaison de ces éléments avec l'amélioration de la qualité des soins (respect de la vie privée du patient, temps d'attente, formation du prestataire), et ou le suivi des femmes enceintes pour qu'elles utilisent l'établissement de santé pour l'accouchement. Les stratégies qui ont été trouvées pour accroitre l’offre des soins post-partum comprennent l'amélioration de la qualité des soins, l'identification au niveau communautaire et la référence des problèmes post-partum et le programme de bons de transport.

**Conclusions:**

Pour accélérer les réalisations en matière d'accouchement institutionnel et d’offre de soins post-partum en Afrique subsaharienne, nous recommandons des stratégies qui peuvent être mises en œuvre de manière durable ou produire des changements durables. La manière de motiver durablement les acteurs communautaires dans les interventions sanitaires peut être particulièrement importante à cet égard. En outre, nous recommandons que davantage d'études d'intervention soient mises en œuvre en Afrique de l'Ouest et du Centre, et qu'elles soient davantage axées sur les soins post-partum.

## Background

Every day, nearly 830 women die from preventable causes related to pregnancy or childbirth globally [1]. Most (99%) maternal deaths occur in developing countries [2], and 65% of these deaths occur in sub-Saharan Africa [3]. Achieving the Sustainable Development Goal target of reducing the global maternal mortality ratio to less than 70 deaths per 100,000 live births by 2030 remains challenging in sub-Saharan Africa [4].

Maternal mortality ratios are highest where skilled birth attendance is the lowest [3]. Most maternal deaths occur during the intrapartum and peripartum periods [5, 6] emphasizing the importance of timely access to quality health services for birth and postpartum care. Furthermore, complications during childbirth and postpartum, such as haemorrhage, pre-eclampsia, or obstructed labour, postpartum infections, are best managed and treated in well-equipped and staffed health facilities. Yet maternal health service coverage varies across sub-Saharan Africa, with facility births ranging from 22% in Chad to more than 90% in the Democratic Republic of the Congo, Gabon, Malawi, and Rwanda [7]. Similarly, the proportion of women receiving postpartum care within two days following birth ranged from 16% in Ethiopia to 84% in South Africa in 2016 [8].

Increasing the proportion of women delivering in facilities and receiving postpartum care has been the focus of numerous interventions globally, including in sub-Saharan Africa [9–12]. These interventions vary in terms of context, content, actors, level of implementation, duration and outcomes assessed, resulting in differential outcomes within sub-Saharan Africa. Such intervention strategies and their effects on increasing facility births and provision of postpartum care in the sub-Saharan African context exclusively have not been systematically reviewed. Systematic reviews have reported on interventions aiming to increase facility births or postpartum care in the global context [13, 14]. However, these reviews did not disaggregate findings by region or by intervention strategy. Since context will affect strategies’ implementation and effectiveness, more in-depth insight is required regarding the different intervention strategies implemented in sub-Saharan African countries specifically, and their effects [15, 16]. This is crucial, to inform contextualized programming and policies for improving facility births and provision of postpartum care for sub-Saharan African mothers, with the overall goal of reducing maternal morbidity and mortality.

The objective of this scoping review is to describe the characteristics and effectiveness of interventions targeting increased facility birth or provision of postpartum care in sub-Saharan Africa (Additional file 1).

## Methods

Colquhoun et al. have defined a scoping review as a form of knowledge synthesis that addresses an exploratory research question, aimed at mapping key concepts, types of evidence, and gaps in research related to a defined area of field by systematically searching, selecting, and synthesizing existing knowledge [15]. It differs from a systematic review, which summarizes the results of carefully designed healthcare studies (such as controlled trials) and provides a high level of evidence on the effectiveness of a healthcare intervention [16].

The present scoping review followed a pre-specified protocol reviewed by relevant senior researchers (TD, AD, BdK, LB and AME), and conducted in line with Arksey and O’Malley’s framework and stages for the conduct of scoping reviews, combined with the Levac et al.’s enhancement of scoping study methodology [15]. The stages include identification of the research question, identification of relevant studies, study selection, data charting or extracting, results collating, summarizing and reporting. This review is reported according to the PRISMA checklist for scoping reviews (Additional file 2) [17].

### Identification of research questions

To address the main objective of the review, we sought to answer the following research questions: (i) what is known from the existing literature on interventions implemented for improving coverage of facility birth and or postpartum care in sub-Saharan Africa? and (ii) What is known from the existing literature on the effects of such interventions on facility births or provision of postpartum care in sub-Saharan Africa?

By addressing these questions, we sought to map the range of interventions intended to improve coverage of facility birth and/or postpartum care in sub-Saharan Africa overall, and by the level of success of intervention characteristics in effect on facility birth or postpartum care.

### Identification of relevant studies

We searched for systematic reviews, scoping reviews, qualitative and quantitative studies using experimental, quasi experimental, or observational designs, which reported on interventions for increasing facility birth or provision of postpartum care in sub-Saharan Africa. These studies were published in English or French. We searched six scientific literature databases (Pubmed, EMBASE, CINAHL, les Revues et Ouvrages en Sciences Humaines et Sociales (CAIRN), la Banque de Données en Santé Publique (BDSP), and the Cochrane Library). We also used grey literature through a search on Google Scholar and snowball or citation tracking (reviewing reference lists of included studies for additional relevant articles). Combination of the study key words and their synonyms were used to search eligible studies from the grey literature. The most recent search was done on 7^th^ October 2019.

The literature search was carried out by the principal investigator (BSC). The search strategy was reviewed by TD, AD, BdK, LB and AME. All selected references were saved in Mendeley desktop software.

### Operational definitions

Operational definitions of key concepts are presented in Table 1.

**Table 1:** Operational definitions of key concepts

Concept	Definition
Sub-Saharan African countries	We followed the UN Development Program definition of sub-Saharan Africa which includes 46 countries, listed by region: East Africa (Tanzania, Kenya, Uganda, Rwanda, Burundi, South Sudan, Eritrea, Ethiopia, Madagascar, Malawi, Mozambique) West Africa (Benin, Burkina Faso, Cape Verde, The Gambia, Ghana, Guinea, Guinea-Bissau, Ivory Coast, Liberia, Mali, Mauritania, Niger, Nigeria, Senegal, Sierra Leone and Togo) Middle Africa (Angola, Cameroon, Central African Republic, Chad, Congo Republic, Democratic Republic of Congo, Equatorial Guinea, Gabon, and Sao Tomé & Principe) Southern Africa (Botswana, Lesotho, Namibia, South Africa, Swaziland, Zambia, and Zimbabwe) Indian ocean (Mauritius, Seychelles)
Facility birth	Any birth after 28 weeks of pregnancy occurring in a health facility
Postpartum care	Any maternal health care within the first six weeks (42 days) following birth, by a health professional, within or outside of a health facility [18]
Intervention	The level of implementation (e.g. community or health system level), the intervention target (e.g. pregnant women, husbands, health providers, etc.), the package implemented, the mode of implementation, the actors implementing the package, and the frequency and duration of implementation
Intervention effectiveness	We considered an intervention as effective if it increased facility births or provision of postpartum care as reported by the authors

### Study selection

**Fig. 1:**
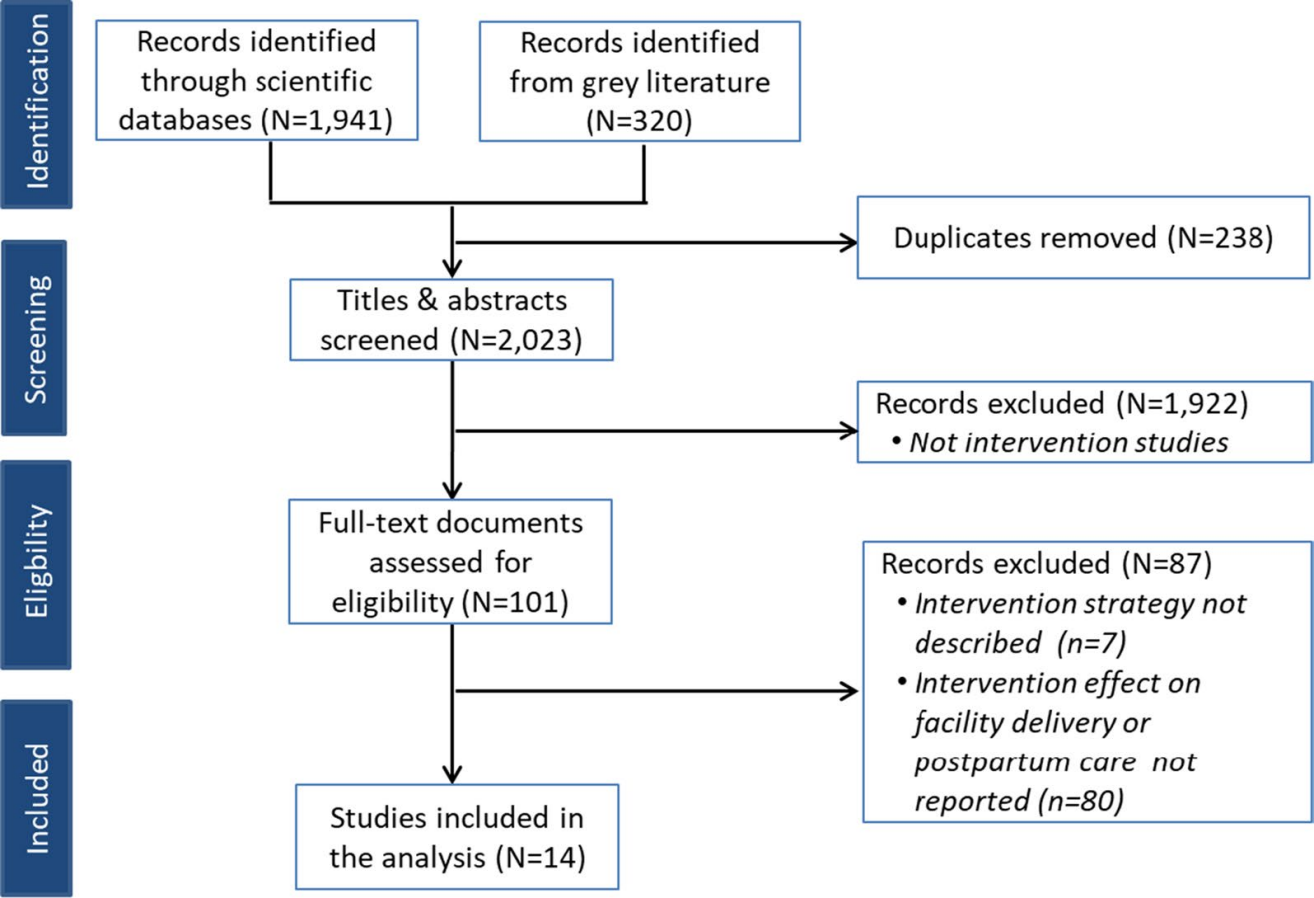
Flow chart of the review records selection

The selection process included identification, screening, and eligibility checks (Fig. 1) [19]. At the identification stage, all records identified from the scientific and grey literature databases were combined and duplicates were removed. We then screened the titles and abstracts and studies which did not report on an intervention were excluded. We then extracted the full-text of all articles and assessed their eligibility using the following selection criteria: (a) described the intervention strategy, including at least the level of implementation, the intervention package, and the intervention actors; (b) reported the effect of the intervention on facility birth or postpartum care coverage. The screening and eligibility checks were conducted in duplicate by BSC and AD. Discrepancies at the two stages were then discussed and solved.

### Data extraction

Data were extracted by the two independent reviewers (BSC & AD) using an Excel data extraction form. The form was tested on five articles before use. The data collected included macro-data or descriptive characteristics of selected studies (authors, year of publication, the program/project that implemented the intervention, country, area of the country (rural or urban), title, objective, study design, year of data collection, whether intervention effect on facility birth is reported, whether intervention effect on postpartum care is reported), and micro-data or analytical data. This included level of implementation *(community level, health system level)*, intervention actors, intervention description, intervention frequency, definition of postpartum care, and intervention effect. We intended to extract information on intervention sustainability and authors’ recommendations regarding future interventions, but were unable to include this in the analysis since studies reported such information rarely.

### Data analysis and synthesis

We conducted a descriptive analysis. We attempted to highlight what elements of the interventions the study authors reported as key to achieving the intervention aims, and which ones were not. Study characteristics, intervention characteristics and intervention effects as reported by the authors were summarized and presented in tables by intervention level and according to whether they targeted facility birth or postpartum care.

## Results

### Characteristics of studies included in the review

**Fig. 2:**
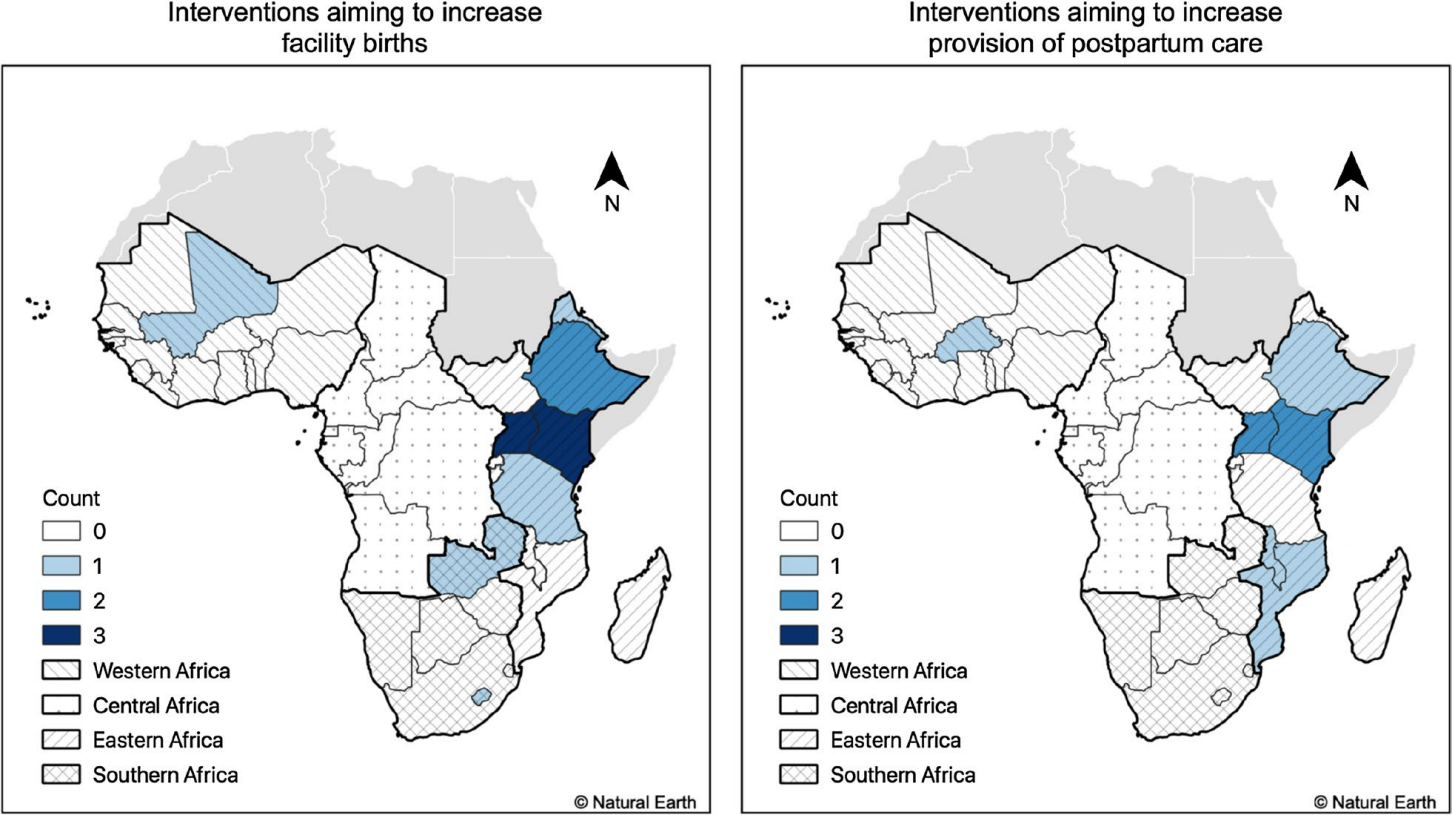
Distribution of interventions aiming to increase facility births and provision of postpartum care in sub-Saharan Africa, 2007–2019

We screened 2023 unique studies in title and abstract and assessed 101 in full text. Most of the studies excluded in full text screening did not report on the intervention effect on facility births or postpartum care (n = 80) or the intervention strategy (n = 7). Fourteen studies published between 2007 and 2019, meeting full inclusion criteria were included in this review (Fig. 1 and Table 2). Nine studies were conducted in East Africa (Kenya (n = 3) [20–22], Uganda (n = 2) [23, 24] Tanzania (n = 2) [9, 25], Eritrea (n = 1) [26], and Ethiopia (n = 1) [27]). Two were conducted in Southern Africa (Zambia (n = 1), [28], and Lesotho (n = 1) [29]), and one in West Africa (Mali) [30]. One multi-country study [31] included countries in East Africa (Kenya, Malawi, Mozambique), and West Africa (Burkina Faso). Nine studies reported on interventions targeting facility birth; two studies reported on three distinct interventions targeting provision of postpartum care [9, 31], and three studies targeting both facility birth and provision of postpartum care [20, 24, 32]. Distribution of selected interventions according to whether they aim to increase facility births or provision of postpartum care, is mapped in Fig. 2. Nine studies used a quasi-experimental design [20, 22–25, 30, 32–34]. Four studies were cross-sectional [21, 29, 31, 35]. In terms of assessment methods, two studies used mixed methods [21, 31].

**Table 2:** Characteristics of studies included in the analysis

Authors	Year of publication	Country	Area	Implementing agency	Year of data collection	Study design	Outcome(s) assessed	Definition of postpartum care	Effect estimate
Ommeh et al. SPS:refid::bib^23^[23]	2019	Kenya	[Not reported]	PharmAccess Foundation	[Not reported]	Cross-sectional mixed methods study	Facility birth	Not applicable	Proportion difference
Altaye et al. [37]	2018	Ethiopia	Rural	The Last Ten Kilometers (L10 K) Project	12/2014–01/2015	Cross-sectional study measuring the effects of self-reported exposure to Family Conversation strategy	Facility birth	Women receiving care at home by Health Extension Workers at within the 48 h following delivery	Proportion difference
Djelouli et al. [33]	2017	Burkina Faso, Kenya, Malawi, Mozambique	Rural	The Missed Opportunities in Maternal and Infant Health (MOMI) Project	10/2015–01/2016	Qualitative study using realist evaluation	Provision of postpartum care	Not reported	Qualitative insights
Masub-Saharan Africavon et al. [26]	2017	Uganda	[Not reported],	Doctors with Africa CUAMM	[Not reported]	Quasi-experimental study	Facility birth Provision of postpartum care	Women receiving care at a health facility after delivery	Proportion difference
Pallangyo et al. [36]	2017	Tanzania	Urban	collegial facilitation intervention project, ‘Improving postpartum care’ (IPPC)	2015 and 2016	Mixed methods study with the quantitative part using a pre-and-post evaluation	Provision of postpartum care	Not reported	Qualitative insights
August et al. [27]	2016	Tanzania	Rural	Ministry of Health and Social Welfare	2012 and 2014	Quasi-experimental study	Facility birth	Not applicable	Proportion difference
Wang et al. [30]	2016	Zambia,	Rural	Ministry of Health	2013	Cluster randomized trial	Facility birth	Not applicable	AOR
Wilunda et al. [34]	2016	Ethiopia	[Not reported]	Doctors with Africa CUAMM	2013 and 2015	Pre-and-post evaluation study	Facility birth Provision of postpartum care	Women receiving care at a health facility after delivery within the seven days following delivery	AOR Proportion difference
Mwaniki et al. [24]	2015	Kenya	Rural	District Health Management Team	2011 and 2012	Pre-and-post evaluation study	Facility birth	Not applicable	Difference in proportion
Obare et al. [22]	2013	Kenya	Rural	Ministry of Health	2010	Quasi experimental study	Facility birth Provision of postpartum care	Care up to six weeks after delivery	OR Proportion difference
Satti et al. [31]	2012	Lesotho	Rural	Partners In Health (PIH)	2009 and 2011	Cross-sectional study	Facility birth	Not applicable	Monthly average number
Turan et al. [35]	2011	Eritrea	Rural	Ministry of Health, UNFPA, Stanford Eritrean Women’s Health Project	2005 and 2007	Quasi experimental study	Facility birth	Not applicable	Proportion difference
Sangho et al. [32]	2010	Mali	Rural	Centre de recherche d’études et de documentation pour la survie de l’enfant (CREDOS)	2007 and 2008	Intervention study using pre-and-post evaluation	Facility birth	Not applicable	Proportion difference
Mbonye et al. [25]	2007	Uganda	Rural and urban	[Not reported]	2003 and 2005	Non-randomized community trial	Facility birth	Not applicable	Proportion difference

*AOR* adjusted odd ratio, *OR* odd ratio

### Interventions for increasing facility births

All twelve interventions aiming to increase facility births were reported as successful at increasing facility births. Eight were implemented at both community and health system levels [20–22, 24, 28–30, 32], whereas four were implemented only at the community level [23, 25–27]. All interventions included community actors and five also included health providers [22, 28–30, 32]. The different intervention characteristics and effects are summarized in Table 3, and described by intervention level.

**Table 3:** Interventions for increasing facility births in sub-Saharan Africa

Authors, year, Country	Community level intervention					Health system level intervention					Intervention effect
	Target	Package	Mode	Actors	Time period	Target	package	Mode	Actors	Frequency	
Altaye et al., 2018, Ethiopia [37]	Pregnant women & family decision makers	Awareness-raising: ANCs, nutrition & birth preparedness	Conversation, distribution of booklets	CHWs	Twice during pregnancy						63% (N = 688) in the intervention group versus 56% (N = 3502) in the comparison (p = 0.011)
August et al., 2016, Tanzania [27]	Pregnant women	Awareness raising: ANC, birth preparedness, postpartum danger signs	Individual home sessions using pictorial cards	CHWs	At least four times during pregnancy?						75.6% (N = 603) to 90.2% (671) in the intervention group versus 76.1% (N = 591) to 79.6% (N = 588) in the comparison group [no p-value reported]. Difference in difference: 11.5% points increase (p = 0.123)
Turan et al., 2011, Eritrea [35]	Women and men	Awareness raising: ANC, birth preparedness, birth danger signs	Group sessions	Maternal health volunteers	Once a week						3% (N = 7) to 47% (N = 58) in intervention group versus 4% (N = 9) to 15% (N = 31) in the comparison group; p = 0.003
Mbonye et al., 2007, Uganda [25]	Pregnant women	Awareness raising: malaria, ANC, administration of malaria preventive treatment	Home visits	TBAs, drug vendors, CHWs, peer mobilizers	Twice						From 34.3% (N = 276) before the intervention to 41.5% (N = 434) after the intervention, p = 0.02
Ommeh et al., 2019, Kenya [23]	Pregnant women	Ambulance voucher for delivery	Enrollment	Community health volunteers	Once	Pregnant women, ambulance drivers	Ambulance voucher for delivery	Enrollment during ANC & training	[Not reported]	Once	In Suguta area: 98% (N = 52) after the intervention versus 30% (N = 16) before the intervention In Barsaloi area: 100% (N = 33) after the intervention versus 30% (N = 10) before the intervention
Massavon et al., 2017, Uganda [26]	Pregnant women	Awareness raising about the voucher program	Radio and stakeholders meetings	Community volunteers, health providers	[Not reported]	Pregnant women	Transport voucher for delivery	Distribution during ANC	[Not reported]	[Not reported]	20% [N not reported] to 80% in the intervention group versus 44% [N not reported] to 61% [N not reported] in the comparison group that is a difference in difference of 43% higher in intervention group
Masavon et al., 2017, Uganda [26]	Pregnant women	Awareness raising about the baby kit	Radio and stakeholders meetings	Community volunteers, health providers	[Not reported]	Pregnant women	Baby kits (plastic basin, soap, polythene bag, ½ kg of sugar, a piece of cotton cloth for wrapping the baby)	Distribution during ANC	[Not reported]	[Not reported]	47% [N not reported] to 58% [N not reported] in the intervention group versus 90% [N not reported] to 71%[N not reported] in the comparison group that is a difference in difference of 30% higher in intervention group
Wang et al., 2016, Zambia [30]	Pregnant women	Promotion about the baby kit	[Not reported]	Community volunteers	[Not reported]	Pregnant women	Baby kit (cloth, baby diaper, blanket)	Promotion during ANC; distribution after delivering in health facility	Health providers	[Not reported for promotion]; Once (for distribution)	36.3% (N = 359) in the intervention group versus 26% (N = 304) in the comparison group (AOR: 1.63; P-value < 0.01, 95% CI: 1.29; 2.06)
Mwaniki et al., 2015, Kenya [24]	Pregnant women	Tracing and accompanying women to health facility for delivery	Household visits	TBAs, CHWs	Routinely	Pregnant women	Pregnancy screening; ANC follow up; payment for services in installments; reduction of waiting time, iron & ARVs available, enhancement of patient privacy; dialogue with community	During routine activities	Health providers	Routinely	52% (N = 259) after intervention versus 33% (N = 164) before the intervention, p = 0.012
Obare et al., 2013, Kenya [22]	Pregnant women	Voucher for maternal health expenses	Identification of women through a poverty grading tool	Distributors from a voucher management agency	Once	Pregnant women	Provision of standard quality services	Routine services	Health providers	Routinely	63% (N = 453) in the intervention group versus 37% (N = 118) in the comparison group; OR:2.1 (95% CI: 1.5 – 3.1)
Satti et al., 2012, Lesotho [31]	Pregnant women	Awareness raising on facility-based care; identification and accompaniment of women to the health center and back home after delivery	Community gatherings (awareness raising); household visits (identification)	NGO Partners in Health, maternal health workers	Once a month	Pregnant women	HIV testing & treatment, nutrition support to babies, baby kits for women delivering in health facility; maternal waiting house	Routine services	NGO Partners in Health, health providers, maternal health workers	Routinely	16 women on average delivering each month in the 2^nd^ year of the intervention, compare to 12 women in the 1^st^ year of the intervention and 4 women in the year preceding the intervention
Sangho et al., 2010, Mali [32]	TBAs, pregnant women	Training on delivery and resuscitation, equipment for TBAs (gloves, plastic sheets, bleach, soap, compresses) identification and awareness raising of pregnant women	Session (training), [Not reported for the others]	CHWs, women’s associations, [Not reported for the others]	Once for training and equipment, [Not reported for awareness raising]	Health providers, pregnant	Training on delivery and resuscitation, equipment, care provision	Session (training), routine services (care)	Health providers for care, [Not reported for training]	Once for training and equipment, routinely for care	*Dialakoroba*: 93% (N = 243) after the intervention versus 86% (N = 324) before the intervention (*p* = 0.005) *Safébougoula:* 90% (N = 271) after the intervention versus 80% (N = 304) before the intervention ( *p* = 0.0005)
Wilunda et al., 2016, Ethiopia [34]	CHWs, village leaders, community	Strengthening sensitization capacity; promotion of free maternal services	Radio broadcasts for promotion, [Not reported for the others]	[Not reported]	[Not reported]	Local health system managers, hospital, health centers, pregnant women	Improving infrastructures, information system, meeting coordination, monitoring; removal of user fees, equipment of maternity wards, training and supervision of CHWs, free ambulance services, care provision	Standard checklists for supervision, routine services for care, [Not reported for the others]	Doctors with Africa, hospital staff (training), health providers (care)	Routinely for care, [Not reported for the others]	60% [N not reported] during the intervention compared to 27% [N not reported] before the intervention; AOR: 5.04 (2.53–10.06)

*AOR* adjusted odd ratio, *OR* odd ratio

#### *Community-level interventions (n* = *4)*

Altaye et al. described an intervention including household-based awareness-raising sessions on antenatal care (ANC), nutrition, and birth preparedness (the need to start saving money for childbirth;, knowledge about danger signs during pregnancy; how to act if an emergency occurs) delivered by community volunteers in rural Ethiopia. The sessions targeted pregnant women together with family members who potentially play a role in decision-making (such as her husband, her mother, mother in-law, elder sisters) [27]. As a result, 63% (N = 688) of pregnant women exposed to the intervention delivered in a health facility compared to 56% (N = 3502), in the non-exposed group (p = 0.011). Turan et al. and August et al., respectively, reported similar intervention characteristics such as ANC, birth preparedness, and birth danger signs as health education topics with men and women through group sessions by community volunteers in Eritrea [26] and with pregnant women through individual household-based sessions facilitated by incentivized community health workers (CHWs) in Tanzania, respectively [25]. Facility births increased from 3% (N = 7) to 47% (N = 58) in the intervention group versus 4% (N = 9) to 15% (N = 31) in the comparison group (p = 0.003) in Eritrea. In Tanzania, facility births increased from 76% (603/798) to 90% (671/744) in the intervention group versus 76% (598/786) to 79% (588/742) in the comparison group [no p value reported]. In Uganda, Mbonye et al. reported that household-based awareness raising sessions with pregnant women focused on malaria and ANC along with administration of malaria preventive therapy [23]. The sessions were led by traditional birth attendants (TBAs), drug shop vendors, community reproductive health workers, and adolescent peer mobilizers. This increased facility births from 34 to 42% (p = 0.02).

#### *Combined health facility and community-level interventions (n* = *8)*

Quality improvement of maternal health services and community-level follow up on women were reported to yield good results as well. In Kenya, Mwaniki et al. reported that health care providers, through a peer discussion process, identified the factors hindering provision of quality maternal health services in their settings, as well as solutions to address them [22]. For instance, ANC clients were followed up through phone calls, women were allowed to pay for laboratory tests in installments, waiting time during ANC was reduced by removing ‘unnecessary’ steps; privacy was enhanced during birth by using a dedicated room and restricting entrance. At the community level, TBAs were entrusted with identifying and accompanying women to the health facility for childbirth. The authors reported that the intervention increased facility birth from 33 to 52% (p = 0.012). In Mali, Sangho et al. implemented an intervention in two health districts (Dialakoroba and Safébougoula). Health workers and TBAs were trained on childbirth care and resuscitation, CHWs and women’s associations were entrusted with communicating with pregnant women for behavior change and following them up until childbirth [36]. As a result, facility births increased from 86 to 93% (p = 0.005) in Dialakoroba and from 80 to 90% (p = 0.005) in Safébougoula.

Voucher programs were also found to be successful. In Kenya, health expenses vouchers were distributed to economically disadvantaged women seeking maternal and reproductive health services [20]. Voucher program coverage was associated with a two-fold increase in delivering at a health facility (OR: 2.1; 95% CI: 1.5–3.1). Also in Kenya, vouchers were distributed to women in two districts (Suguta and Barsaloi) to cover the costs of hospital ambulance transportation to the health facility for childbirth [21]. As a result, 98% (N = 52) in Suguta and 100% (N = 33) in Barsaloi delivered at a health facility compared to only 30% (N = 16 and 10, respectively) in the control groups [no statistical comparison performed]. In Uganda, a transport voucher program increased facility births from 20 to 80% in the intervention sub-county versus 44% to 61% in the comparison sub-county [no statistical comparison performed] [24].

Non-monetary interventions, alone or combined with other strategies, appeared to have positive effect as well. In Uganda, baby kits composed of plastic basin, soap, polythene bag, ½ kg of sugar, and a piece of cotton cloth for wrapping the baby, were offered to women who attended health facility for birth or postpartum care [24]. This increased facility birth coverage from 47 to 58% in the intervention group compared to a decrease from 90 to 71% in the comparison group [no statistical comparison performed]. Likewise, in Zambia, offering a baby kit composed of baby clothes, baby diaper and a blanket to every woman attending a health facility for childbirth was found to increase the likelihood of giving birth in a health facility (AOR: 1.63, 95% CI 1.29–2.06) compared to not offering any kits to mothers [28]. In Lesotho, women delivering at health facility were offered a baby kit; and women completing all ANC visits were tested for HIV, and if necessary, provided with HIV treatment and support for the baby’s nutrition [29]. A maternity waiting house (in the health facility) accommodated women from remote villages during the two weeks preceding the expected birth date. In addition, community gatherings were organized to increase people’s awareness about facility birth; incentivized community maternal health workers identified and accompanied women to the health center for ANC, childbirth and postpartum visits. Reported findings of this intervention showed a monthly average number of 16 women delivering in a health facility during the 2nd year of intervention, compared to an average of 12 women in the 1st year of intervention, and an average of 4 women in the year preceding the intervention [no statistical comparison performed].

Local health system and community actor strengthening, along with removal of user fees for maternal health services, increased facility delivery in Ethiopia [32]. Indeed, a local health management office was constructed and furnished, its health information and coordination systems strengthened, user fees removed for emergency obstetric care at the hospital, health centers equipped and supplied with drugs and consumables, health providers trained, referral system strengthened, health extension workers’ and village leaders’ capacity was strengthened in community awareness-raising on maternal and child health. The authors reported that as a result, women in the intervention zone were five times more likely to deliver in a health facility than women in the comparison zone (AOR: 5.04 (95% CI 2.53–10.06)).

### Interventions for increasing provision of postpartum care

Six interventions [20, 24, 31, 32, 34] were designed to increase provision of postpartum care, half of which were reported as successful [24, 31, 34] (Table 4). Five interventions were implemented at both the health system and community levels [20, 24, 31, 32], and one only at the health system level [34]. All these interventions targeted women delivering in facilities or at home. Two interventions targeted postpartum care provided at the health facility and at home [31, 34], while the four others targeted postpartum care as provided in health facility.

**Table 4:** Interventions for increasing provision of postpartum care in sub-Saharan Africa

Authors, year, Country	Community level intervention					Health system level intervention					Intervention effect
	Target	Package	Mode	Actors	Frequency	Target	Package	Mode	Actors	Frequency	
Pallangyo et al., 2017, Tanzania [36]						Health providers	Training on facilitation skills, supervision, national postpartum care guidelines, priority setting for improving postpartum care	Participatory workshop	Peer trained health providers	[Not reported]	Health providers testified about the increased number of mothers now attending postpartum care: *‘’The number of mothers who are coming for postpartum has gone high…’’*. Mothers were reported to sensitize their husbands about postpartum care at health facility: *‘’I have observed great awareness among community members, because even men are accompanying their wives to the [postpartum care] clinic.’’*
Djellouli et al., 2017, Burkina Faso, Kenya, Malawi, Mozambique [33]	CHWs & postpartum women	For CHWs: training on postpartum care, promotion of breastfeeding, and FP counseling; For women: postpartum care, referrals of postpartum complications, FP counseling, promotion of breastfeeding	Individual & group sessions for promotion, [Not reported for the others]	CHWs for care, referrals, promotion; [Not reported for training]	[Not reported]	Health providers & postpartum women	For providers: training on postpartum & FP care; For women: postpartum care, FP services, promotion of breastfeeding	Clinical mentorship and quality of care reviews (for the training in Malawi), [Not reported for the others]	Health providers for care & promotion; [Not reported for training]	[Not reported]	The bridging function of CHWs between community and health facility increased uptake of postpartum care: CHWs’ sense of responsibility and motivation was due to community’s trust in them, their sense of belonging to the health facility (training, supervision, and incentives), the visible signs of their connection to the formal sector (uniforms, institution’s bicycles). The visibility of the ‘bridge’ between CHWs and the health facility reinforced community trust and established connectivity. In Burkina Faso, positive talks by women about the ‘bridge’ increased provision of postpartum care. Health providers’ motivation to provide postpartum care was not related to the training received, but to an existing pay-for-performance system
Massavon et al., 2017, Uganda [26]	Pregnant women	Awareness raising about the voucher program	Radio and stakeholders meetings	Community volunteers, health providers	[Not reported]	Pregnant women	Transport voucher for delivery	Distribution during ANC	[Not reported]	[Not reported]	1% [N not reported] to 49% [N not reported] in the intervention group versus 13% [N not reported] to 12% [N not reported] in the comparison group that is a difference in differences of 49% higher is the intervention group
Massavon et al., 2017, Uganda [26]	Pregnant women	Awareness raising about the baby kit	Radio and stakeholders meetings	Community volunteers, health providers	[Not reported]	Pregnant women	Baby kits (plastic basin, soap, polythene bag, ½ kg of sugar, a piece of cotton cloth for wrapping the baby)	Distribution during ANC	[Not reported]	[Not reported]	3% [N not reported] to 26% [N not reported] in the intervention group versus 8% [N not reported] to 33% [N not reported] in the control group that is a difference in differences of 2% lower in the intervention group
Obare et al., 2013, Kenya [22]	Pregnant & postpartum women	Voucher for maternal health expenses	Identification of women through a poverty grading tool	Distributors from a voucher management agency	Once	Pregnant and postpartum women	Provision of standard quality services	Routine services	Health providers	Routinely	73% (N = 649) in the intervention group versus 61% (N = 274) in the comparison group; OR: 1.3 (95% CI: 0.9 – 1.8)
Wilunda et al., 2016, Ethiopia [34]	CHWs, village leaders, community	Strengthening sensitization capacity; promotion of free maternal services	Radio broadcasts for promotion, [Not reported for the others]	[Not reported]	[Not reported]	Local health system managers, hospital, health centers, pregnant women	Improving infrastructures, information system, meeting coordination, monitoring; removal of user fees, equipment of maternity wards, training and supervision of CHWs, free ambulance services, care provision	Standard checklists for supervision, routine services for care, [Not reported for the others]	Doctors with Africa, hospital staff (training), health providers (care)	Routinely for care, [Not reported for the others]	34% [N not reported] during the intervention compared to 30% [N not reported] before the intervention; AOR: 1.02 (0.60–1.73)

*AOR* adjusted odd ratio, *OR* odd ratio

#### *Health facility and community level- interventions (n* = *5)*

Two of the five health system and community level interventions were reported as successful. Djellouli et al. reported that postpartum services were improved in quality at health facility level and decentralized to community level in Burkina Faso, Kenya, Malawi and Mozambique [31]. Health providers and incentivized CHWs were trained to provide postpartum care, family planning (FP), and promote breastfeeding to mothers. CHWs referred cases of postpartum complications to the health facility. The findings showed that the ‘bridging’ function of CHWs between communities and health facilities increased uptake of postpartum care. First, CHWs’ sense of responsibility and motivation for this function was due to community’s trust in them, their sense of belonging to the health facility (training, supervision, incentives), the visible signs of their connection to the formal sector (uniforms, institution’s bicycles, use of pictorial checklist). Second, the visibility of the collaboration between CHWs and the health facility reinforced community trust in the facility and established connectivity. In Burkina Faso, women talked positively to each other about CHWs’ connection to the health facility and the care provided there. These positive talks were favored by community leaders and linked to the good care experience at health facility. The intervention reported by Massavon et al. was the transport vouchers program covering maternal health service users in Uganda [24]. It was reported to lead to an increase in postpartum care coverage from 1 to 49% in the intervention group, while there was a small decrease from 13 to 12% in the comparison group.

Three interventions were deemed unsuccessful for increasing provision of postpartum care even though they were found successful in increasing facility births. First, the baby kit (plastic basin, soap, polythene bag, ½ kg of sugar, a piece of cotton cloth for wrapping the baby) incentive program in Uganda reported by Massavon et al. was found to result in a slight (2%) reduction in use of postpartum care in the intervention group compared to the control group [24]. Second, Obare et al. reported that 73% of women who benefitted from the voucher program for maternal health services in Kenya received postpartum care compared to 61% among those who did not benefit from the program (AOR: 1.3, (95% CI 0.9–1.8)) [20]. Wilunda et al. also found that the health system and community actor strengthening program in Ethiopia did not increase provision of postpartum care (34% during the intervention versus 30% before the intervention; AOR: 1.02 (0.60–1.73)) [32].

#### Health system level intervention

The one health system-level intervention addressing postpartum care, reported by Pallangyo et al., consisted of improving postpartum care quality through health provider training in Tanzania [34]. The qualitative findings suggest that the intervention may have been successful: health providers mentioned about the increased number of mothers attending postpartum care: *‘’the number of mothers who are coming for postpartum has [increased]…’’*; mothers were also reported to discuss postpartum care at health facility with their husbands.

## Discussion

The present review contributes to filling out knowledge gap in the existing literature regarding utilization of maternal health services in sub-Saharan Africa. Interventions targeted at increasing facility births include single-component strategies, specifically community awareness raising, reduction of health expenses (vouchers, removal of user fees), non-monetary incentive programs, and multi-component strategies that combine the aforementioned components with other components such as improvement of service quality, or follow-up on pregnant women to use health facility for birth. Strategies that were reported to increase provision of postpartum care included improvement of quality of care, community-level identification of women and referrals of postpartum problems and a transport voucher program.

Reviews on interventions targeting increased facility births and postpartum care have already been reported. Cochrane reviews by Lassi et al. and Mbuagbaw et al. in 2015 showed that community level and health system interventions can increase the number of women giving birth in health facilities [14, 39]. However, these reviews did not specify the effect of the increase per intervention package. What’s more they did not exclusively target sub-Saharan Africa. A review by Belemsaga et al. in 2015 reported that women’s discussion groups to identify perinatal problems and formulate strategies had a high impact on the prevalence of women using postpartum care [40]. However, this intervention was not implemented in sub-Saharan Africa. The present scoping review fills in these knowledge gaps by reporting which intervention package produces what effect on health service utilization for childbirth and postpartum care in sub-Saharan African settings. Such information is more contextual and specific in terms of intervention package, thereby can better contribute to guiding further research or intervention scale up in sub-Saharan African regions.

In this review, all interventions designed to increase facility births were reported as successful. This is promising given that further progress regarding facility birth rates needs to be made in sub-Saharan Africa. Facility births have drastically increased in many sub-Saharan African settings over the last decade, but not equally across the board with some settings (rural) or social groups (low income groups) lagging behind [3]. Moreover, the maternal mortality ratio requires further reduction in sub-Saharan Africa and facility birth is considered a major way of achieving that. However, this can only be achieved when quality of facility birth including quality of care as perceived by women (women’s experience), is improved.

However, it is important to note that these findings might not be generalizable to the whole of sub-Saharan Africa, since most of the interventions were implemented in Eastern or Southern Africa, leaving a gap in regions such central and western Africa. The disparity in study distribution across sub-Saharan African regions mirrors another distinction- between Anglophone (eastern and southern Africa) and Francophone (western and central Africa) parts of the continent. Such disparity could therefore be an artifact of the literature available since most of sub-Saharan Africa studies in the literature are published in English and from Anglophone countries [37]. Such research disparity between Anglophone and Francophone countries in Africa could be enforced by the concentration of funded research in Eastern and Southern Africa [38]. One future research priority could therefore be to undertake research targeting interventions to increase facility birth and postpartum care in Western and Central African regions. What’s more, few studies included in this review (n = 6) targeted postpartum care, as compared to twelve studies targeting facility birth. There is need to undertake more research on intervention targeting increased postpartum care in sub-Saharan Africa.

Furthermore, for policy makers to be better guided about potential intervention strategies, they should be informed not only about intervention effectiveness, but also about intervention sustainability to understand how well for instance incentives, vouchers or user fee removal interventions could be expanded and sustained in the sub-Saharan African context. For instance, where various studies have shown that users continue to make informal payments for maternal health services despite user fee exemption policies [39, 40]. Calls for overcoming sustainability of health promotion programs have been increasing [41, 42]. In fact, social scientists have emphasized the challenge of sustainable intervention models that aim to change organizational practices since settings are adaptive and dynamic, making it often difficult to get the ‘rules of the game’ to change [42]. We therefore recommend as a future research priority to assess sustainability of interventions targeting increased facility births or postpartum care, using appropriate study designs such as randomized control trial. Third, it is noteworthy that all twelve interventions identified that targeted facility birth were reported as successful. This finding points toward question the possibility of publication bias, and research outcomes may have influenced authors’ decision or motivation to publish the findings. Yet, facilitating learning from failed interventions is at least as important as sharing insights regarding successful interventions.

There is a need for building intervention models to achieve continuum of perinatal care for mothers and infants in sub-Saharan Africa [43]. Our findings unveil several challenges to build up intervention models to increase facility births and provision of postpartum care in sub-Saharan Africa. First, the variety and complexity of interventions across the different settings, coupled with their uneven distribution across the regions, problematizes conceptualizing an intervention model that could inform locally adapted interventions in sub-Saharan African countries. Second, scaling up costly interventions [20, 21, 24, 29, 32] will be challenging in resources-limited settings. For example, the interventions described by Massavon et al. in Uganda reportedly cost USD 10.5 and USD 9.4 per unit facility birth for the baby kit and voucher packages, respectively. Third, the scarcity of interventions in sub-Saharan Africa to improve postpartum care, and their lack of effectiveness constitute another drawback to proposing intervention models. Despite its critical role for maternal health, postpartum care seems to fall out of the gaze of maternal health researchers, providers and other stakeholders in sub-Saharan Africa [31, 44, 45]. In fact, most maternal and infant deaths occur in the first month after birth, almost half occurring within the first 24 h, 66% during the first week [46]. With the urgent need to guide heath policies in sub-Saharan Africa to reduce maternal morbidity and mortality [3], this paper calls researchers and other stakeholders to test less costly and simplified interventions, further examining intervention adaptation and undertaking more intervention studies that target increased provision of postpartum care to develop a strong evidence base.

Lastly, we found that the majority (12 out of 15) of the included interventions involved actors from the community, engaging in community awareness raising and mobilization, but also bridging activities such as medical follow-up during pregnancy, timely health facility referrals, outreach maternal counselling and postpartum care. The WHO recommends community participation in maternal health interventions [47], and our review supports that community actors are key to not only improving maternal health indicators, but also for strengthening local health systems through implementing maternal health programs. However, sustainability of CHWs’ involvement is questionable; motivating community actors and coordinating their activities have implications for community health actors. Different health system programs, NGOs and researchers tend to have these community actors on board for their respective and (often) concomitant activities. Establishing local coordination systems, e.g. at the district level, to regulate CHW involvement in any health intervention could make their workloads more manageable and prevent possible conflicts of interest between health interveners. Second, we found that community actors were involved in different health interventions as volunteers [21, 26–28] or as employees [29, 31]. Paying community actors is a known source of motivation for their work [31]; not paying CHWs puts intervention sustainability at stake in low-resource settings. What is needed is greater recognition that CHWs are not cost free. Additionally, it is important mentioning that no study included took into account sociocultural considerations in designing and assessing the interventions. Yet, countries included in this review are culturally diverse. Insights on cultural considerations could better highlight differences and similarities of interventions and their effects across sub-Saharan African countries or regions. This would enlighten more future interventions targeting increased facility births or postpartum care. Hence, we recommend that future similar researches take into account sociocultural dimensions of the interventions.

This review seeks to stimulate further implementation research to improve maternal health indicators in sub-Saharan Africa. Strengths of our review include the scoping review design, which did not restrict our exploration to a given study design or information sources. However, it has some limitations. First, some of the studies included did not fully describe the implemented interventions, e.g. missed information on the actors involved or the frequency of intervention package implementation. This makes description and replication of these strategies challenging. Second, given the design of this study—scoping review—no assessment of study quality was done within the review. This limited our ability to estimate intervention effectiveness. To make evidence synthesis of intervention effectiveness easier, we recommend authors of future studies use statistical tests for comparison, compare outcomes with control groups, and fully report statistical information on intervention effect, including the count of observations (n), the proportion, mean or median in the groups being compared, the effect measure (e.g. odd ratio, risk difference), and the probability value. Third, the studies included had different designs, meaning they used different methods to assess intervention effectiveness. Systematic reviews—including studies with similar designs—could better summarize intervention effect. However, this requires sufficient numbers of eligible studies meeting systematic review quality criteria. Finally, it is hard to generalize the findings of this review to all of sub-Saharan Africa because the interventions did not cover all the regions of sub-Saharan Africa, nor were implementation sites and regions necessarily representative of the respective countries.

## Conclusions

Strategies reported to improve facility birth coverage in the sub-Saharan African context include community awareness raising, health expense reduction, non-monetary incentive programs such as provision of baby kits (plastic basin, soap, bag, blanket), or a combination of these with improvement of service quality (privacy, waiting time, training of providers) and/or follow-up of pregnant women to use health facility for birth. Strategies that were found to improve postpartum care coverage include improvement of care quality, community-level identification and referrals of postpartum problems and transport voucher program.

To better contribute to increasing facility births and provision of postpartum care in sub-Saharan Africa, we recommend strategies that can be implemented sustainably or produce sustainable change. Moreover there is a need to undertake more intervention studies in West and Central Africa, and develop and evaluate more postpartum care interventions. Finally, we encourage more reflection on how to sustainably motivate community actors in health interventions.

## Supplementary information

**Additional file 1.** Article search strategies.

**Additional file 2.** Preferred Reporting Items for Systematic Reviews and Meta-Analyses extension for Scoping Reviews (PRISMA-ScR) Checklist.

## Data Availability

All studies included in this review and data extracted from these studies are available and accessible upon authorization of co-authors. To access these data, please send a request to bienvenusalimcamara@gmail.com.

## Abbreviations

ANC: Antenatal care
AOR: Adjusted Odd Ratio
CHW: Community Health Worker
FP: Family planning
NGO: Non-Governmental Organization
TBA: Traditional Birth Attendant
WHO: World Health Organization
