# Supplementary material for: Interventions to increase facility births and provision of postpartum care in sub-Saharan Africa: a scoping review
Source: Reprod Health. 2021 Jan 21;18:16. doi: 10.1186/s12978-021-01072-4 (PMC7819232; doi:10.1186/s12978-021-01072-4)
Supplement: Supplementary file 1 — Additional file 1. Article search strategies. [file 12978_2021_1072_MOESM1_ESM.docx]

**Additional file 1. Search strategy**

| **Scientific databases** | **Syntax** |
| --- | --- |
| PubMed | *(("methods"[MeSH Terms] OR "methods"[All Fields] OR "intervention"[All Fields]) OR implementation[All Fields]) AND ((facility[All Fields] AND ("delivery, obstetric"[MeSH Terms] OR ("delivery"[All Fields] AND "obstetric"[All Fields]) OR "obstetric delivery"[All Fields] OR "delivery"[All Fields])) OR (institutional[All Fields] AND ("delivery, obstetric"[MeSH Terms] OR ("delivery"[All Fields] AND "obstetric"[All Fields]) OR "obstetric delivery"[All Fields] OR "delivery"[All Fields])) OR (facility[All Fields] AND ("parturition"[MeSH Terms] OR "parturition"[All Fields] OR "birth"[All Fields])) OR ("postnatal care"[MeSH Terms] OR ("postnatal"[All Fields] AND "care"[All Fields]) OR "postnatal care"[All Fields] OR ("postpartum"[All Fields] AND "care"[All Fields]) OR "postpartum care"[All Fields]) OR (("postpartum period"[MeSH Terms] OR ("postpartum"[All Fields] AND "period"[All Fields]) OR "postpartum period"[All Fields] OR "postpartum"[All Fields]) AND visit[All Fields]) OR ("postnatal care"[MeSH Terms] OR ("postnatal"[All Fields] AND "care"[All Fields]) OR "postnatal care"[All Fields]) OR (postnatal[All Fields] AND visit[All Fields])) AND "sub-saharan Africa"[All Fields]* |
| Cochrane Library | *"implementation" in Title Abstract Keyword OR "intervention research" in Title Abstract Keyword AND "facility delivery" in Title Abstract Keyword OR "institutional delivery" in Title Abstract Keyword OR "Africa" in All Text* |
| BDSP | *(intervention) OR (implementation) AND (facility birth) OR (facility delivery) OR (accouchement) OR (postpartum) OR (postnatal) OR (Sub-Saharan Africa)* |
| CAIRN | *(intervention OR implementation) AND (birth OR postpartum OR postanatal) AND sub-saharan Africa* |
| Grey literature |  |
| Google scholar | *("implementation research") AND ("institutional delivery" OR "facility birth" OR "facility delivery" OR "postpartum care" OR "postpartum visit") AND "sub-Saharan Africa"* |
